# Impact of Father Involvement and Positive Parenting on Child Mental Health: Insights From a Survey of Ugandan Households

**DOI:** 10.1111/famp.70144

**Published:** 2026-04-15

**Authors:** Ronald Asiimwe, Tim Welch, Lekie Dwanyen, Rosco Kasujja, Firminus Mugumya, Adrian J. Blow, Kadija Mussa

**Affiliations:** ^1^ Department of Family Social Science University of Minnesota Twin Cities Minnesota USA; ^2^ Department of Human Development and Family Science Oklahoma State University Stillwater Oklahoma USA; ^3^ Department of Human Development and Family Studies Michigan State University East Lansing Michigan USA; ^4^ Department of Mental Health Makerere University Kampala Uganda; ^5^ Department of Social Work and Social Administration Makerere University Kampala Uganda

**Keywords:** child mental health, father involvement, father positive parenting, Uganda

## Abstract

The influence of father positive parenting and involvement on children's mental health outcomes is underexplored in many sub‐Saharan African countries, such as Uganda, despite research showing that fathers play a critical role in shaping their children's mental and emotional health outcomes. Most research on father involvement in parenting has been conducted in high‐income countries in Western countries, and most research from Africa relies on mothers' reports. This study surveyed 236 Ugandan fathers raising children aged 6–17 years on their parenting and their children's mental health issues. Using the *Mplus* software, we conducted path analysis to predict child mental health symptoms (attention, internalizing, and externalizing) with father involvement and father positive parenting as independent variables while also controlling for the covariates. Results indicated that father involvement was negatively associated with attention problems (*β* = −0.28, *p* < 0.001), internalizing problems (*β* = −0.11, *p* = 0.02), and externalizing problems (*β* = −0.50, *p* < 0.001). Conversely, father's positive parenting had a small but statistically significant association with only internalizing problems (*β* = −0.11, *p* = 0.03). Further, we conducted exploratory analyses to examine whether marital status influenced these associations. We found that father involvement was negatively associated with externalizing and attention symptoms among married and unmarried fathers. Conversely, positive parenting was not significantly associated with internalizing, externalizing, or attention symptoms in either group. These findings suggest that greater father involvement may reduce behavioral and emotional issues in Ugandan children and thus emphasize the need to involve more fathers in parenting interventions.

## 1 Introduction

Father involvement and father positive parenting are critical determinants of child mental health outcomes. While extensive research on father involvement in parenting has been conducted in mostly high‐income countries in Western contexts (e.g., Coates and Phares 2019; Fitzgerald et al. 2020; Lamb 2000), there is a growing recognition of the need to understand the contributions and role of fathers in child caregiving within culturally diverse, low‐resource settings such as Uganda (e.g., Garcia et al. 2022; Siu et al. 2017). In line with this growing area of research, this study explores the impact of father involvement and positive parenting on child mental health in Ugandan households, considering the unique socio‐cultural and economic factors that shape parenting practices in this context.

In recent years, there have been increased efforts to understand and improve parenting practices in Uganda, such as parental warmth and support, positive parenting, and child discipline, given research highlighting their associations with children's overall well‐being (e.g., Boothby et al. 2017; Walakira et al. 2021). This shift has led to greater investment in culturally adapted parenting programs aimed at strengthening parenting practices to improve child outcomes (Amollo et al. 2022; Byansi et al. 2024; Siu et al. 2024; Wieling et al. 2017). However, despite this growing body of work, few studies have specifically examined the impact of fathers' positive parenting and father involvement on child mental health, even though research suggests that paternal engagement plays a critical role in children's psychological and emotional well‐being (Diniz et al. 2021). Existing studies on fatherhood in Uganda have primarily focused on father involvement in relation to children's academic outcomes (Mahuro and Hungi 2016; Mugumya, Mwesigye, and Ahimbisibwe 2023, Mugumya, Karooro, and Mwesigye 2023), maternal and child health (Muheirwe and Nuhu 2019), social norms shaping father's caregiving practices (e.g., Nnyombi et al. 2025), and child protection outcomes such as reduced incidences of abuse and maltreatment (Ashburn et al. 2017; Siu et al. 2017). While these contributions are important, they do not fully capture how fathers' involvement and use of positive parenting practices influence children's mental health outcomes.

To guide our discussion of father involvement in Uganda, we drew on Lamb et al.'s (2017) biosocial framework of involvement, which conceptualizes *father involvement* (defined as the amount of time fathers spend with their child and the degree of responsibility they assume for their children) as a multidimensional construct comprising three interrelated dimensions: interaction, availability and responsibility. *Interaction* refers to the more intense and direct, one‐on‐one father's interactions with the child whether through caretaking (e.g., feeding, dressing) or shared activities (e.g., playing with the child, helping with schoolwork, and others). *Availability* concerns the father's potential availability for interaction with the child, by virtue of being present or accessible to the child whether or not direct interaction is occurring (Lamb et al. 2017). Availability could include activities that require less intense degrees of direct interaction with the child (e.g., the father cooking in the kitchen while the child plays in the room next). The final dimension is *responsibility* which is the “extent to which the parent takes ultimate responsibility for the child's welfare and care” (Lamb 2000, 31), including decision‐making related to health, education, and daily caregiving. Among the three dimensions of involvement, Lamb et al. (2017) contends that *responsibility* is the most complex to define yet arguably the most critical. It extends beyond direct interaction with the child to include arranging childcare, planning supervision, and ensuring children's material and emotional needs are met. More fundamentally, responsibility reflects a father's capacity for anticipation, sensitivity, and contingency planning.

Lamb's biosocial framework of father involvement builds on a long historical evolution of how fatherhood has been conceptualized. According to Lamb (2000), the late 19th‐ and early 20th‐century perspectives on fathers largely shaped by religious, philosophical, and medical discourses, portrayed fathers primarily as moral guides. With increased industrialization, the Great Depression, and World War II, the breadwinner role for fathers became the dominant marker of paternal competence. In the 1930s and 1940s, critiques of fathers' effectiveness as role models, particularly for sons, led to the conceptualization of fathers as sex‐role exemplars. By the 1970s, the new nurturant father ideas emerged, emphasizing that beyond breadwinning, role modeling, and moral guidance/teaching, fathers needed to be more emotionally present and actively engaged in caregiving for their children, in addition to fulfilling the traditional roles.

In summary, Lamb's tripartite framework, comprising of these three dimensions captures both visible and less observable dimensions of fathering, and thus has over the years demonstrated these historical shifts. Contemporary research indicates that while fathers on average interact less with children than mothers, their involvement varies across ecological, social, and individual factors, including motivation, skills, and institutional opportunities (Lamb et al. 2017). Given the above context, we deemed Lamb's framework to be particularly relevant in our exploration of father involvement in Uganda, where fatherhood is similarly experiencing some shifts in terms of what the father's roles are perceived to be by society. In contemporary Uganda for example, fathers remain expected to provide, protect, and discipline their children while at the same time are increasingly called upon to offer emotional support, co‐parent, and participate in daily childcare (Nnyombi et al. 2025). Therefore, by disaggregating involvement into three distinct but interrelated dimensions, the framework allowed us to engage in a more nuanced, culturally and contextually grounded examination of how different forms of paternal involvement might influence the various target children's mental health outcomes.

In this study, we define father positive parenting as fathers' consistent use of nurturing, supportive, and developmentally oriented behaviors that promote children's emotional security, competence, and well‐being. It is crucial to understand that within the Ugandan context, positive parenting encompasses not only emotional warmth, responsiveness, and guidance, but also *instrumental caregiving practices* (e.g., ensuring school attendance, providing educational supplies, and supporting skills development) that signal a father's care, commitment, and investment in children's futures. These instrumental tasks have been backed by research from Uganda as culturally appropriate and meaningful indicators of positive parenting in Ugandan households (Boothby et al. 2017). By adopting this broader, culturally responsive definition, the study captures how fathers express support and involvement in ways that align with local caregiving norms and realities.

Given the evolving family dynamics in Uganda, the important role of fathers in Ugandan households, and the significant socioeconomic disparities (e.g., poverty, disease burden, natural disasters, and exposure to war and organized violence), which continuously shape parenting and family processes, there is a pressing need for research that centers fathers' roles in supporting child mental health. Understanding the unique contextual factors that influence paternal involvement in Uganda can inform culturally responsive interventions that strengthen family systems and promote child well‐being.

### 1.1 The Context of Fatherhood and Caregiving Arrangements in Uganda

In Uganda, fatherhood has long been regarded as a symbol of pride, prestige, and a marker of maturity (Kironde and Addis 2023). Traditionally, being a father carries the expectation of serving as the household head, with overarching authority to provide basic needs, discipline children, and care for the family, roles deeply embedded in Ugandan cultural norms (Nabugoomu et al. 2020). Within this patriarchal structure, father involvement has often been defined primarily in terms of economic provision, with less emphasis on emotional engagement, and participation in other caregiving roles. Ugandan families also tend to function within extended family systems, where caregiving responsibilities are shared among relatives such as grandparents, aunts, and uncles, and in some instances with community members (e.g., neighbors; Visser 2024). While this communal caregiving system can provide a critical support network to buffer against socioeconomic hardships, it can also dilute direct father–child involvement, as parenting duties are distributed across multiple caregivers.

Additional structural challenges further complicate the involvement of fathers in caregiving roles. For instance, labor migration, particularly in rural areas, often results in the physical absence of fathers, hindering father–child bonding and sometimes negatively impacting children's mental health outcomes (Ferrone and Giannelli 2015). A 16‐study review conducted in 2021 by AfriChild, an interdisciplinary research center at Makerere University, highlighted common cultural expectations of fatherhood in Uganda, which included: ensuring safety and protection, providing for basic needs, instilling discipline and good morals, and ensuring children's future through education. These responsibilities underscore the high societal value placed on father provision but also reveal how fatherhood is framed largely through obligations rather than relational or emotional roles.

Qualitative studies have provided further insight into how Ugandan men experience fatherhood. Kironde and Addis (2023), in a study with 24 Ugandan men, found that fathers derived joy and fulfillment from meeting financial obligations, but they also reported emotional strain and psychological pressure when unable to provide. This raises questions about how the stress associated with provision‐focused fatherhood may affect children's long‐term mental and emotional wellbeing. Research in post‐conflict Northern Uganda suggests that while discipline remains a central parenting function, fathers are increasingly recognizing the value of nurturing and emotionally supporting their children. Mehus et al. (2018) reported that paternal emotional involvement acted as a protective factor against the negative psychological effects of war‐related trauma among children, underscoring the importance of fathers' supportive presence in contexts of adversity.

Other empirical studies from Uganda point to broader developmental benefits of father involvement and positive parenting. For example, Kyazze et al. (2020) investigated the parenting practices of 100 Ugandan fathers and found significant positive correlations between paternal engagement and children's curiosity (*r* = 0.40, *p* < 0.05), learning (*r* = 0.42, *p* < 0.05), and creativity (*r* = 0.38, *p* < 0.05). Fathers who encouraged exploration and play helped children develop cognitive and creative skills, suggesting that father involvement extends beyond discipline and provision to foster developmental growth.

Despite these insights, most existing research on parenting and child mental health in Uganda has disproportionately focused on mothers (Möllerherm et al. 2024; Nyqvist and Jayachandran 2017; Singla and Kumbakumba 2015). This imbalance often overlooks the critical role fathers play in shaping children's emotional and psychological well‐being. In a context where men face considerable cultural pressure to provide, protect, and lead households (Mathur et al. 2016), examining the implications of father involvement and positive parenting is essential for a more holistic understanding of family processes, well‐being, and child development.

Recent shifts in cultural narratives highlight evolving perceptions of exemplary fatherhood in Uganda. Beyond financial provision, contemporary discourses increasingly emphasize fathers' emotional engagement and supportive roles (Kironde and Addis 2023; Mehus et al. 2018). Coupled with growing mental health awareness campaigns across the country, this evolution underscores the importance of studying father involvement and positive parenting not only as a marker of masculinity and cultural identity but also as a determinant of children's mental health outcomes. By examining father positive parenting and involvement in relation to children's internalizing, externalizing, and attention problems, this study contributes to filling a critical gap in research and informs the design of culturally responsive family mental health interventions in Uganda (Siu et al. 2024).

### 1.2 The Current Study and Research Questions

The primary aim of this study is to investigate the relationships between father positive parenting and involvement on children's internalizing, externalizing, and attention problems among children in Uganda. The current study specifically answers the following research questions: Among Ugandan children, how is fathers' positive parenting and father involvement associated with internalizing, externalizing, and attention problems? (RQ1) Are covariates such as the father's marital status, level of education, and number of children associated with internalizing, externalizing, and attention problems in children (RQ2). We hypothesize that father involvement and father's positive parenting would both predict lower levels of reports of attention problems, internalizing and externalizing problems in children.

## 2 Method

### 2.1 Procedures and Design

Data for this study was gathered using a survey‐based approach. Institutional approval was obtained from two research institutions, Makerere University in Uganda (the study setting) and Michigan State University (the first author's institution at the time of data collection). Participants were recruited through local online parenting groups and public places frequented by parents and caregivers (e.g., healthcare centers and places of worship). Inclusion criteria were being 18 years or older, with the ability to provide informed consent; being a father or caregiver of a child or children 6–17 years of age; and being a native of Western Uganda, fluent in either English or Runyankole. Eligible participants signed informed consent forms by hand before participating in the study. Participants completed, by hand, a comprehensive survey in groups of 10–20 that gathered at community health centers located in participant areas of residence. To ensure consistency and comparability in reporting children's symptoms, fathers with more than one child were asked to select one focal child based on two criteria: (a) the child with whom they had the most distant relationship, or (b) the child they perceived as experiencing the most significant behavioral and emotional health challenges. Data was collected in person by the first author and two graduate research assistants. The researchers read the questions aloud and guided participants in selecting the response categories that best reflected their parenting behaviors. All items and response categories were thoroughly explained to participants at the beginning of each session. All study questionnaires were translated into the local of the study setting, Runyankole, by expert translators and then back translated by an independent bilingual expert, with discrepancies reviewed and resolved through consensus to ensure linguistic accuracy and cultural appropriateness. To stay focused on the main aims of this particular study, we were unable to provide additional detail regarding the translation and adaptation process of the APQ measure used in this study; however, interested readers may refer to another article by Asiimwe et al. (2025), which provides a detailed account of the translation and cultural adaptation procedures.

### 2.2 Participants

The sample were 236 Ugandan fathers raising children aged 6–17 years. We targeted this broad age range for two reasons: (a) to reflect Ugandan family realities in which parents often have multiple children at varying developmental stages under their care and (b) because it covers critical developmental stages from middle childhood to adolescence. During these periods, children experience significant cognitive, emotional, and social changes, making them particularly vulnerable to mental health challenges (Sawyer et al. 2018). Research shows that father involvement has a profound impact on shaping children's mental, emotional, and behavioral well‐being during these stages (Fitzgerald et al. 2020). Most fathers (86.7%; *n* = 202) were married, 4.7%; *n* = 11 were either widowed or divorced, and 8.6%; *n* = 20 were single or never married. The majority (62.7%; *n* = 148) of fathers resided in rural areas, 28.8%; *n* = 68 urban areas, and 8.1%; *n* = 19 between rural and urban areas. In terms of religious affiliation, 91.9%; *n* = 215 identified as Christian, 7.7%; *n* = 18 Muslim, and 0.4% did not identify their religious affiliation. Most fathers (76.6%; *n* = 180) had lower grades than a bachelor's degree, 22.1%; *n* = 52 had a bachelor's degree, and two fathers (0.9%) had a master's degree. Reported socioeconomic status (SES) was 58.5%; *n* = 137 low income, 32.9%; *n* = 77 lower‐middle‐income, 7.7%; *n* = 18 high‐income, and 0.9% other. Most fathers (53.4%; *n* = 125) had 1–3 children, 30.8%; *n* = 72 had 4–5 children, and about 15.4%; *n* = 36 had 5 or more children in their care. Data related to the number of children was missing for one father out of the 236.

### 2.3 Measures and Data Collection

#### 2.3.1 Participant Demographics

Standard and relevant participant demographics were gathered using a demographic form which was part of the comprehensive questionnaire completed by participants. Data collection took place throughout the year 2022.

#### 2.3.2 Independent Variables

##### 2.3.2.1 Father's Involvement

Three items from the involvement subscale of the Alabama Parenting Questionnaire adapted for use in Uganda (in Asiimwe et al. 2025) were used to assess father involvement. These items were: “You volunteer to help with special activities that your child is involved in (e.g., sports, dance groups, church youth groups),” “You engage in, or do fun activities (e.g., jump a rope, tell stories, go to parties, or visit friends) with your child,” and “You calmly explain to your child why his/her behavior was wrong when he/she misbehaves.” Responses to these items were on a five‐point Likert scale from “*never*” (1), “*almost never* (2),” “*sometimes* (3),” “*often* (4)”, and “*always*” (5). For this analysis we summed up the individual items to create a scale. The scores ranged from 3 to 15. Because Cronbach's alpha may not be accurate when using ordered categorical data with five or fewer response options (Flora 2020), we used McDonald's Omega (McDonald 1999) to estimate reliability, with a *⍵* = 0.69, and an *M* = 10.59 (SD = 2.30).

##### 2.3.2.2 Father Positive Parenting

Similarly, four items from the same adapted Uganda APQ measure assessing positive parenting were used to assess father's positive parenting. The items on the scale were: “You teach or ensure that your children have technical skills (e.g., washing clothes, making baskets and mats, cooking, sowing clothes, milking cows etc.),” “You ensure that your child arrives to school on time,” “You ensure that your child has school supplies (e.g., books, pens, pencils etc.),” and “You attend PTA meetings, parent/teacher conferences, or other meetings at your child's school.” Responses on the positive parenting scale were also on the same five‐point Likert scale used to measure father involvement. Scores ranged from 4 to 20, with an *⍵* = 0.73 and *M* = 16.82 (SD = 2.09). As earlier noted, it is important to note the relevant cultural context of Uganda that is unique in terms of culturally appear indicators of positive parenting. Some items on our positive parenting scale capture instrumental and technical tasks, such as ensuring school attendance or providing learning materials, and others. These behaviors were included because they represent culturally relevant expressions of support and investment in children's well‐being in the Ugandan context. In a nutshell, instrumental caregiving in many Ugandan settings often co‐occurs with warmth and responsiveness, and both contribute to fostering children's development.

#### 2.3.3 Child Outcome Variables

##### 2.3.3.1 Externalizing, Internalizing, and Attention Problems

The outcome variables were assessed using items from the 17‐item Pediatric Symptom Checklist (PSC) which has been culturally adapted for use in another sub‐Saharan Africa setting (Lowenthal et al. 2011). The PSC organizes symptoms into three subscales: *internalizing problems* (e.g., “Your child worries a lot”), *externalizing problems* (e.g., “Your child fights with other children”), and *attention problems* (e.g., “Your child gets distracted easily”). Parents respond to items on each subscale using a 3‐item Likert scale: *never* (0), *almost never* (1), and *sometimes* (2). We used McDonald's Omega to measure reliability, with an *⍵* = 0.71 (internalizing), *⍵* = 0.87 (externalizing) and *⍵* = 0.70 (attention).

#### 2.3.4 Covariates

We also included measures of parental self‐report of socioeconomic status (low‐income, lower‐middle‐income, and high‐income), parental employment, level of education, marital status, and the number of children present in the home and whether the children are biologically related to the parent.

### 2.4 Plan of Analysis

The *Mplus* software version 8.2 (Muthén and Muthén 2017) was used for statistical analysis, with full information maximum likelihood estimation to account for missing data. To compute McDonald's ⍵, we used R Studio (Version 1.3) and the *psych package* (Revelle 2015). All variables were measured as manifest variables, using summed scores. Initial analyses tested associations using structural equation modeling, with both the exogenous and endogenous variables measured as latent constructs. The weighted least squares and variance adjusted (WLSMV) was used to account for the categorical nature of the data. However, this resulted in an analysis with multiple solutions, and the log likelihood failed to replicate. Increasing the number of starts did not replicate the log likelihood. Because of this, path analysis was used, with all variables measured as manifest variables since manifest‐variable approach can offer greater stability and does not require estimating latent structures or their factor loadings elements that often contribute to estimation difficulties. However, the significance of the parameters was similar across both analyses. The sole exception was the association between father's positive parenting and internalizing symptoms.

**FIGURE 1:**
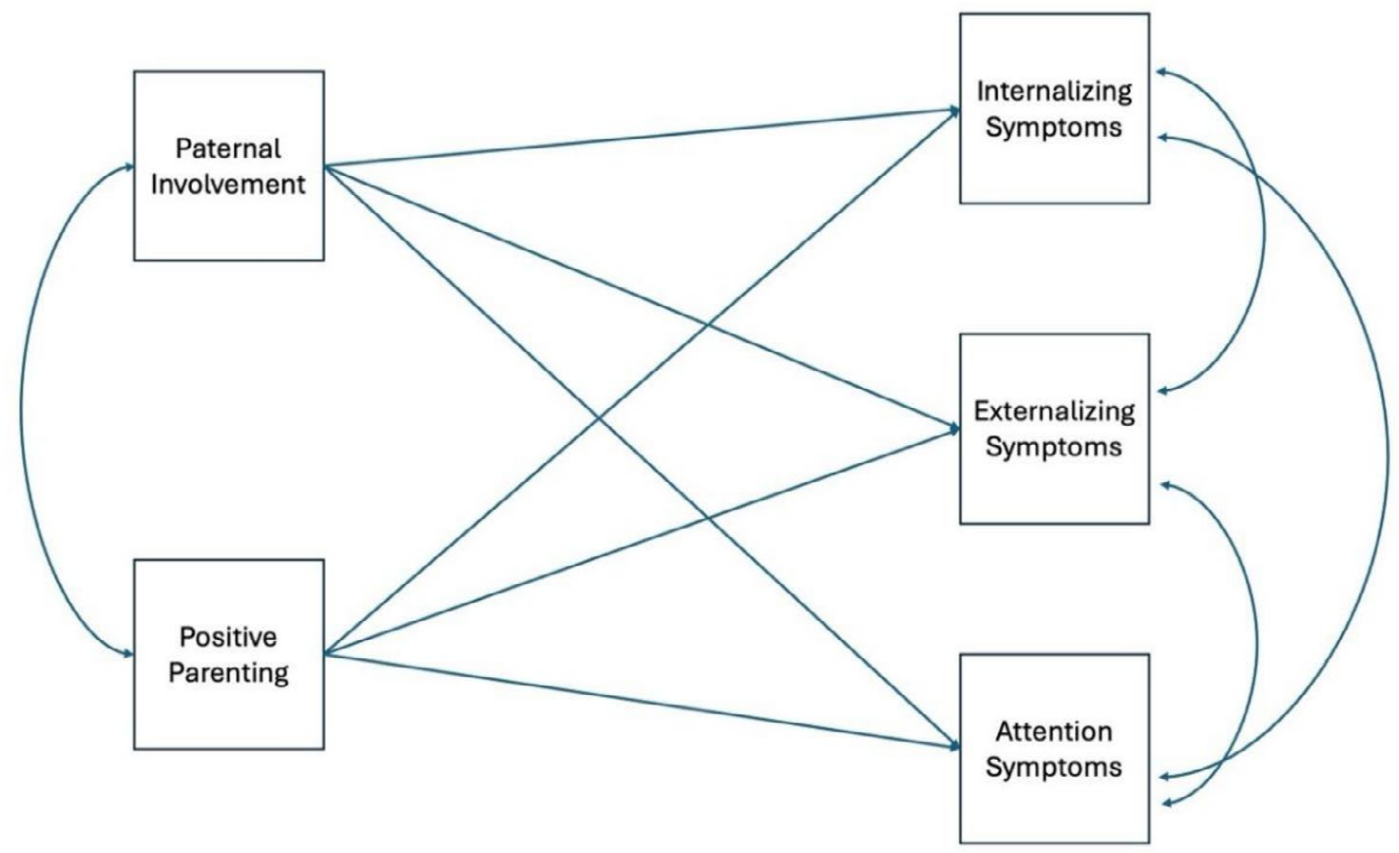
Hypothesized path analysis model of influence of father, father's positive parenting and father's involvement on child internalizing, externalizing, and attention problems.

Path analysis was used to predict child mental health symptoms (attention, internalizing and externalizing) with father involvement and father positive parenting as independent variables, while also controlling for the covariates. Figure 1 presents a conceptual diagram of the path analysis. For simplicity, the covariates are not included in the diagram. Because a path analysis is just identified with zero degrees of freedom, it has perfect model fit, so fit indices are not reported.

## 3 Results

### 3.1 How Does Father Involvement Predict Child Internalizing, Externalizing, and Attention Problems?

**FIGURE 2:**
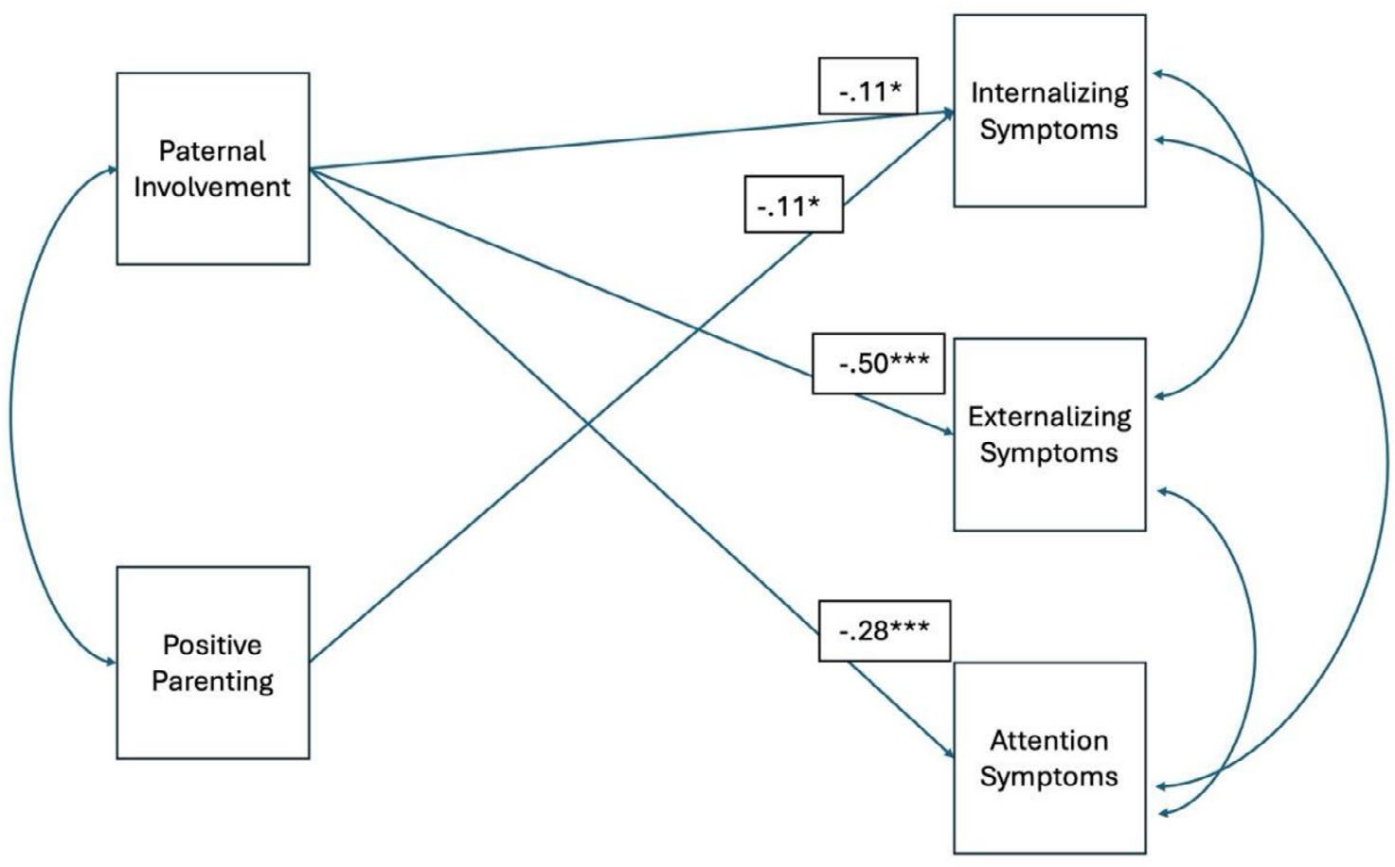
A display of a significant pathway (standardized beta). *p < 0.05, ***p < 0.001.

We found that higher father involvement was negatively associated with attention problems (*β* = −0.28, *p < 0*.001), internalizing problems (*β* = −0.11 *p < 0*.05) and externalizing problems (*β* = −0.50, *p* < 0.001; see Figure 2). In this Ugandan context, greater father involvement corresponded with lower levels of attention, internalizing, and externalizing problems. Specifically, for attention problems, a one‐point increase in father involvement was associated with a 0.28 standard deviation decrease, representing a medium effect size. For internalizing problems (e.g., anxiety, depression) every one‐point increase in father involvement corresponds to a 0.11 standard deviation decrease, reflecting a small effect size. Finally, for externalizing problems (e.g., aggression, disruptive behavior) the association was stronger, with a 0.50 standard deviation decrease for every point increase in father involvement.

In summary, greater father involvement was associated with fewer behavioral and emotional problems in children in this Ugandan context, with the strongest effect observed for externalizing problems. The only significant covariate was the father's marital status, which was associated with greater internalizing problems (*β* = 0.23, *p* < 0.05). Specifically, fathers who reported being unmarried (e.g., divorced, widowed, remarried) are more likely to report higher internalizing problems in their children.

### 3.2 How Does Father's Positive Parenting Predict Child Internalizing, Externalizing, and Attention Problems?

There was a small negative and significant association between positive parenting and internalizing problems (*β* = −0.11, *p* < 0.05), but not with externalizing or attention symptoms, as can be seen in Table 1 and Figure 2. For every one unit increase in positive parenting, there is a decrease of 0.11 standard deviations for internalizing problems.

**TABLE 1:** Independent, Dependent, and Sociodemographic Variables Included in the Path Analysis Model.

Parameter	*B*	SE	*p*
Attention			
Father Involvement	−0.28***	0.07	< 0.001
Positive parenting	−0.03	0.07	0.67
Number of children	0.19	0.19	0.32
SES	0.14	0.24	0.55
Employment	−0.08	0.11	0.45
Educational Status	0.1	0.16	0.52
Marital Status	0.08	0.14	0.58
Biological/non‐biological	−0.19	0.19	0.32
Internalizing			
Father Involvement	−0.11*	0.05	0.02
Father Positive Parenting	−0.11*	0.05	0.03
Number of children	0.13	0.13	0.35
SES	0.06	0.17	0.71
Employment	0.02	0.08	0.83
Educational Status	0.12	0.11	0.27
Marital Status	0.23*	0.1	0.02
Biological/non‐biological	0.14	0.13	0.3
Externalizing			
Father Involvement	−0.5***	0.1	< 0.001
Father Positive parenting	−0.09	0.11	0.4
Number of children	0.16	0.27	0.55
SES	0.45	0.34	0.18
Employment	−0.09	0.15	0.54
Educational Status	0.27	0.22	0.23
Marital Status	0.18	0.2	0.36
Biological/non‐biological	−0.45	0.26	0.09

Abbreviation: SES, socioeconomic status.

* *p* < 0.05.

*** *p* ≤ 0.001.

### 3.3 Exploring Potential Moderators Related to Child Outcomes

Because the father's marital status was the only statistically significant covariate associated with internalizing symptoms (*β* = 0.23, *p* < 0.05), we conducted post hoc exploratory multiple‐group analyses to determine if marital status moderated the association between father involvement, father positive parenting and internalizing symptoms. Marital status may shape the broader family environment influencing this association.

#### 3.3.1 Multiple Group Analysis With Marital Status as Grouping Variable

We ran a multiple group path analysis, using marital status as a grouping variable to further explore the statistical significance of marital status as a covariate for internalizing symptoms (Table 2). Because of sample size, we dichotomized marital status into married (*n* = 180) and not married (*n* = 29). We initially included all covariates in our analysis, but doing so resulted in a non‐positive definite first‐order derivative product matrix, which resulted from model non‐identification because there were more parameters estimated than participants in the not married group. Due to this sample size constraint, and because none of the remaining covariates were statistically significant, we ran the analyses without any covariates. The multiple group analysis was otherwise identical to the original path analysis (i.e., both predictor variables and all three outcome variables were included).

**TABLE 2:** Results of Multiple Group Analysis with Marital Status as Grouping Variable.

Parameter	Married (*N* = 180)			Not Married (*N* = 29)		
	B	SE	*p*	B	SE	*p*
Attention						
Involvement	−0.25**	0.08	0.003	−0.54***	0.13	0.001
Positive	−0.05	0.08	0.51	0.22	0.16	0.17
Internalizing						
Involvement	−0.09	0.05	0.08	−0.23*	0.14	0.1
Positive	−0.07	0.05	0.14	−0.08	0.18	0.66
Externalizing						
Involvement	−0.49***	0.11	0.001	−0.65***	0.18	< 0.001
Positive	−0.1	0.11	0.36	0.22	0.16	0.36

* *p* < 0.05.

** *p* < 0.01.

*** *p* ≤ 0.001.

Father involvement was negatively associated with externalizing and attention symptoms in both groups, indicating higher involvement predicted lower symptoms. This finding parallels the findings in the full group analysis. Positive parenting was not significantly associated with internalizing, externalizing, or attention symptoms in either group. This contrasted with the full group analysis which found positive parenting was associated with lower internalizing symptoms. Also, there was not a significant association between father involvement and internalizing symptoms in either group. This finding was also in contrast to the full group analysis which did find a statistically significant negative association, with marital status as the only significant covariate.

To explore the conflicting findings on the protective effect of father involvement for internalizing symptoms in the full group and multiple group analysis, we created an equality test by constraining the association between father involvement and internalizing symptoms. This did not significantly worsen model fit, with a ${\Delta \chi }^{2}$ = 0.92 (1) *p* = 0.34. This demonstrates there is not a significant difference between married vs. not married fathers in the size of the association between internalizing symptoms and father involvement. Interestingly, with the equality constraint, the association became significant in both the married and non‐married groups. To better understand this finding, we conducted a second exploratory analysis using the number of children as a grouping variable. We examined what role the number of children residing in the house may have in the association between internalizing symptoms and father involvement.

#### 3.3.2 Multiple Group Analysis With Number of Children as a Grouping Variable

The results of these analyses can be seen in Table 3. The number of children was chosen as a variable because it may be harder to provide the same level of support (e.g., financial and material) to individual children in a home with many children. Having a larger number of children (i.e., more than four) was relatively common in our sample, with approximately half of fathers (46.2%) reporting having more than four children. We dichotomized the groups into homes with less than four children (*n* = 113) and homes with four or more children (*n* = 97). Because marital status was the only covariate associated with internalizing symptoms, we included marital status as a covariate and included both positive parenting and involvement as well as all three outcomes. For fathers who reported caring for less than four children, there was a significant negative association between father involvement and internalizing symptoms with a (*β* = −0.19, *p = 0*.009). In the group with fewer than four children, marital status was only significantly associated with higher internalizing symptoms (*β* = 0.75, *p* = 0.03) but not externalizing or attention problems. This indicates father involvement is associated with fewer internalizing symptoms for fathers who have less than four children and are married. For fathers who had more than four children, the effect of father involvement on internalizing symptoms was not statistically significant (*β* = −0.07, *p* = 0.31), and marital status was just above the *p* < 0.05 threshold (*β* = 1.13, *p* = 0.051).

**TABLE 3:** Results from Multiple Group Analysis with Number of Children as a Grouping Variable.

Parameter	Less than four children			More than four children		
	*B*	SE	*p*	*B*	SE	*p*
Attention						
Involvement	−0.37	0.11	0.001***	−0.22	0.09	0.012*
Positive	0.09	0.11	0.41	−0.11	0.09	0.21
Marital Status	−0.1	0.54	0.85	0.61	0.77	0.42
Internalizing						
Involvement	−0.19	0.07	0.009***	−0.07	0.06	0.31
Positive	−0.07	0.07	0.35	−0.43**	0.13	0.001
Marital Status	0.75	0.35	0.03	1.13	0.58	0.051
Externalizing						
Involvement	−0.55	0.15	0.001***	−0.43**	0.13	0.001
Positive	−0.02	0.15	0.88	−0.1	0.14	0.48
Marital Status	0.29	0.74	0.70	1.27	1.13	0.26

* *p* < 0.05.

** *p* < 0.01.

*** *p* ≤ 0.001.

Including an equality constraint for the association between internalizing symptoms and father involvement did not worsen model fit with ${\Delta \chi }^{2}$ = 1.61 (1), *p* = 0.20. When we did this, the association became significant in both groups (*β* = −0.12, *p* = 0.01). The association between marital status and internalization remained significant for fathers with less than four children (*β* = 0.86, *p* = 0.01) but was not significant for fathers with more than four children (*β* = 0.39, *p* = 0.60). The association between father's positive parenting and internalizing symptoms was significant for fathers who reported more than four children (*β* = −0.43, *p* = 0.001). This indicates father's positive parenting is associated with fewer internalizing symptoms among fathers who have more than four children.

## 4 Discussion

The study aimed to explore associations between father involvement and father's positive parenting and children's internalizing, externalizing, and attention problems in Uganda. Grounded in Lamb et al. (2017) three‐dimensional biosocial framework, comprising interaction, availability, and responsibility, we found that father involvement was significantly associated with lower reports of internalizing, externalizing, and attention difficulties, even after controlling for relevant demographic variables. Conversely, positive parenting was only negatively associated with internalizing problems in children but not with either externalizing or attention symptoms. Among covariates, only the father's marital status was statistically significant in relation to children's internalizing symptoms.

Globally, studies show that fathers' involvement and father positive parenting have a positive impact on children's mental health and developmental outcomes (Maselko et al. 2019). Our study builds on global and African research showing that fathers' involvement supports children's emotional, behavioral, and developmental outcomes. In South Africa, both parents' involvement reduced adolescents' internalizing and externalizing problems (Profe and Wild 2017). In Ghana, engaged fathers promoted self‐regulation and social competence. In Kenya, father involvement improved cognitive, social, and emotional development in young children (Garcia et al. 2022). Broader international evidence affirms that fathers indeed play a unique and vital role in fostering children's mental health and well‐being (Maselko et al. 2019; Opondo et al. 2016). In Uganda where collectivistic family and cultural values prevail, the father's role is central not only in helping to maintain family cohesion and structure, but also as a protective factor against children's mental and emotional challenges.

The findings of this Uganda study can be interpreted through Lamb et al.'s (2017) biosocial framework dimensions of father involvement, availability, interaction, and responsibility. First, our results found that father involvement was most strongly associated with reductions in children's externalizing problems, such as aggression, defiance, and conduct issues. This aligns with Ugandan cultural norms, where fathers are seen as primary disciplinarians and moral guides. From the *responsibility* dimension of Lamb's framework, it is plausible that Ugandan fathers who actively monitor behavior, provide guidance, and participate in daily routines fulfill critical parental duties, which appears to mitigate externalizing behaviors and support children's social, emotional, and psychological development (Mehus et al. 2018; Siu et al. 2017). Equally, *interaction*, or the quality and father's engagement in daily activities, reinforces positive behavior, fosters resilience, and enhances overall family functioning.

However, for fathers with larger families in our sample, we found that father involvement was not associated with reductions in internalizing symptoms (including depression and anxiety). This may reflect *availability* constraints, as the time and attention fathers can devote to each child could diminish depending on how large the family size is. Together, findings on father involvement underscore that while interaction and responsibility can positively influence children's externalizing outcomes, sufficient availability is necessary to cause a significant effect on children's internalizing symptoms. This further highlights the need for culturally and structurally supportive strategies to optimize father involvement to be able to mitigate the risk of child externalizing, attention, and internalizing problems in Uganda.

Further, Ugandan cultural norms emphasize fathers primarily as financial providers, often resulting in their physical absence due to work obligations (Boothby et al. 2017; Mehus et al. 2018). These dynamic shifts transfer caregiving responsibilities to mothers, older siblings, and extended family members, particularly in larger families where fathers' time and presence are further limited. Consequently, fathers' influence on children's emotional well‐being may be indirect, as larger families often necessitate prioritization of financial support and external discipline over emotional engagement. This may explain why paternal involvement does not consistently correlate with reductions in children's internalizing symptoms, which require sensitivity and personal attention. Similarly, the same argument, related to cultural norms and gendered caregiving roles, could be plausible reasons for why (a) fathers' positive parenting was not associated with externalizing and attention problems as well as (b) why there was a small negative but significant association between father positive parenting and internalizing problems in Ugandan children with the fathers we sampled in our study. As discussed above, in many Ugandan families, caregiving tasks that are often cited as indicators of positive parenting (including playing with child and direct emotional engagement and others) are still socially framed as maternal roles, whereas fathers are primarily seen as providers, protectors, and authority figures, which can limit the expression of positive parenting behaviors in ways that could have a direct effect on children's externalizing and attention problems (Nnyombi et al. 2025). These cultural prescriptions may constrain the frequency or type of father‐child interactions measured as positive parenting, reducing their observable impact on certain behavioral domains.

Another interesting finding of our study was the small negative and significant association between father's positive parenting and internalization problems. Our guess is that this finding reflects a combination of measurement as well as contextual factors rather than weak father's influence per se. Cross‐cultural research (e.g., Lansford 2022) shows that nuanced aspects of parenting, such as emotional warmth and responsiveness, are more consistently linked to internalizing outcomes than broader caregiving tasks that include instrumental support, which may not directly influence children's emotion regulation in the same way, particularly in the Ugandan context. Further, there is meta‐analytic evidence to suggest that broadly, positive parenting effects on internalizing problems are typically small to moderate and context dependent, particularly when measurement does not isolate emotional components specifically tied to internalizing behaviors (Cooke et al. 2022).

Further, although no statistically significant differences were found between married and unmarried fathers regarding associations with children's internalizing symptoms, contextual factors remain critical points of consideration. In Uganda, where marriage is highly valued as a foundation for family structure (Sunder 2019), children of unmarried fathers may experience social stigma, exclusion, and heightened vulnerability to internalizing problems such as anxiety, depression, and emotional distress. In our sample, 31 fathers were unmarried (single or divorced), highlighting a subgroup at potential risk. For unmarried fathers with multiple children, the combination of emotional absence and the demands of providing for a large family may exacerbate neglect and limit their capacity to meet children's emotional needs.

These findings underscore the need to interpret father involvement within Uganda's socio‐cultural and economic context. Family size, marital status, and cultural expectations shape both the availability of fathers and the emotional experiences of children, suggesting that interventions aimed at improving child mental health must consider these nuanced family dynamics.

Finally, perhaps another critical issue of discussion regards our inclusion of a broad age range of children (6–17 years) in the sample and not being able to control for the broad age difference in our analysis. Developmental differences across this age range may influence both father involvement and child mental health outcomes, meaning that the associations observed in this study should be interpreted cautiously. Research indicates that paternal engagement impacts children differently at various developmental stages and affects their emotional and behavioral wellbeing in nuanced ways. Given this context, future studies of similar nature as ours should consider age‐stratified analyses or include interaction terms to clarify these dynamics, enabling more precise, developmentally and culturally informed recommendations for father‐focused interventions.

### 4.1 Implications for Policy, Research, and Practice

Findings from our study underscore the critical role of father involvement in shaping child mental health outcomes in Uganda. Consistent with prior evidence (Mehus et al. 2018), engaged father–child relationships may serve as protective factors against mental and emotional health risks during development. In this study, positive father involvement was associated with reduced internalizing symptoms (e.g., anxiety and depression) but not with externalizing behaviors (e.g., aggression and rule‐breaking) or attention‐related problems. These findings are descriptive and should not be interpreted prescriptively. They suggest that positive parenting, as conceptualized in Western contexts, may not exert uniform protective effects in Uganda. Thus, father involvement should not be pathologized when it diverges from dominant “warm” parenting models. Future research should examine how sociocultural norms and gender dynamics shape paternal practices in Uganda and also broaden definitions of “positive parenting” to include culturally meaningful forms of involvement.

The non‐significant effects of socioeconomic status and employment status of fathers observed in this study may reflect shifting trends and fatherhood norms in contemporary Uganda, where paternal involvement is increasingly being defined not only by breadwinning and discipline (as it was traditionally) but also by the father's emotional presence and relational engagement with children. In Uganda today, Nnyombi et al. (2025) contend that although fathers are still culturally expected to provide economically and exercise authority, contemporary expectations are increasingly emphasizing the fathers' roles in supporting mothers and participating in daily childcare across socioeconomic groups. It is our assumption that this shift may attenuate the direct association between income or employment and child mental health outcomes we witnessed in our study. At the same time, the study also highlights structural barriers that can constrain fathers' engagement, particularly among those from lower socioeconomic backgrounds. Unemployment, long working hours in manual labor, economic strain, and large family sizes may limit fathers' capacity for consistent discipline and emotional attunement, even when relational involvement is culturally valued. Together, these findings underscore the need for culturally and contextually responsive family support programs that move beyond economic indicators to strengthen quality father–child interactions, promote consistent routines, and mobilize community‐based supports. Fathers with larger families may particularly benefit from targeted interventions that mitigate risks associated with limited direct involvement while reinforcing emerging relational models of Ugandan fatherhood.

In the above context, mental health professionals are positioned to integrate discussions of father involvement into therapeutic practice. Given the widespread limited exposure to child development and childrearing knowledge in Uganda (Siu et al. 2017), professional training in culturally appropriate, evidence‐based parenting interventions is warranted. Policymakers should also prioritize father engagement through supportive policies, including parental leave provisions and incentives for men's participation in parenting programs. Together, these efforts may strengthen positive father parenting and involvement and enhance child mental health outcomes in Uganda.

### 4.2 Limitations of the Study

Our study has some limitations. Firstly, since this was not an intervention study, we were unable to assess the effects of an intervention. Consequently, our findings are primarily based on analyzing the direct associations between father involvement, positive parenting, and child mental health outcomes using survey data. Without exploring the direct effects of an intervention, it is challenging to confidently conclude whether father involvement directly affects child mental health or if there are other confounding variables. Second, we relied solely on fathers' reports of their own parenting and their children's behaviors, without incorporating perspectives, such as those from mothers and those of children. It is quite possible that fathers may have overestimated their own behaviors, and disengaged fathers might not have an accurate understanding of their children's behaviors. The inclusion of additional viewpoints from mothers and children could provide much stronger designs and insights.

Third, our participants were recruited through community spaces and online parenting forums with the goal of reaching a diverse pool of fathers across various socioeconomic and regional backgrounds in Uganda. While this approach helped broaden our sample, we acknowledge that it may have inadvertently favored fathers who were more engaged or had better access to community networks, smartphones, and digital platforms. Similarly, our data collection procedure of asking fathers to report on either “the child with whom they had the most distant relationship” or “the child they perceived as having the most behavioral or emotional challenges,” and administering surveys in groups of 10–20 fathers may all have introduced selection bias and social desirability issues, as fathers may have altered responses for fear of judgment by peers despite assurances of confidentiality and independent responding. Together, these factors could have potentially inflated the associations between low father involvement or parenting quality and child difficulties we found in results. Therefore, future research should endeavor to employ mixed and more inclusive recruitment and data collection strategies, such as household‐based sampling and targeted outreach to fathers with limited community or digital access. Such strategies would reduce sampling bias and help ensure that findings reflect the full spectrum of paternal involvement and positive parenting in Uganda, and as well capture fathers across varying engagement levels, socioeconomic circumstances, and caregiving experiences.

Regarding the issue about asking fathers to report on one specific “problematic child,” future research should consider examining all children within a household or using randomly selected focal children to reduce potential selection bias. This approach would allow for a more accurate assessment of how father involvement and positive parenting relate to child mental health across the full range of father–child relationships, rather than focusing only on the most distant or challenging child. Additionally, longitudinal designs could also be helpful in clarifying causal pathways and capturing how fathers' involvement or positive parenting may differentially influence children at varying developmental stages.

Lastly, we also used a single measure of father involvement and positive parenting. However, given the complexity of parenting in LMICs (Garcia et al. 2022), it is highly likely that other types of unmeasured father‐child interactions, such as discipline, monitoring, and supervision, could provide more evidence of the pathways of associations for our findings.

## 5 Conclusion

Our study provides initial evidence that father involvement contributes positively to children's mental health, specifically regarding internalizing, externalizing, and attention difficulties in Uganda. Interestingly, positive parenting was only associated with children's internalizing problems but not with either externalizing or attention symptoms. We also identified that factors such as the father's number of children and marital status had an influence on children's mental health outcomes. Considering ongoing discussions about father involvement in parenting and interventions in Uganda and other low‐ and middle‐income countries (LMICs), this study's results are crucial for integrating fathers' perspectives to inform the adaptation of programs to better meet the needs of fathers in Uganda and similar cultural contexts in Africa.

## Funding

This research was conducted with funding from the Family Process Institute (FPI) dissertation award fellowship; Award No. IP00655814, awarded to the first author as part of his dissertation for his doctoral degree completion from Michigan State University.

## Conflicts of Interest

The authors declare no conflicts of interest pertinent to the subject of this manuscript. Further, the corresponding author (RA) will avail the data files that supported the findings of this research upon reasonable request.

## Data Availability

The data that support the findings of this study are available on request from the corresponding author. The data are not publicly available due to privacy or ethical restrictions.
